# Multiple Intramyocardial Masses in an Otherwise Healthy 35-Year-Old Woman

**DOI:** 10.1016/j.cjco.2021.12.009

**Published:** 2021-12-24

**Authors:** Ramsey G.G. Powell, Frédéric L. Paulin, Jeffrey Flemming, Scott Harris, Stephen A. Duffett

**Affiliations:** a Department of Medicine, Faculty of Medicine, Memorial University of Newfoundland, St. John’s, Newfoundland, Canada; b Department of Nuclear and Molecular Medicine, Faculty of Medicine, Memorial University of Newfoundland, St. John’s, Newfoundland, Canada; c Department of Radiology, Faculty of Medicine, Memorial University of Newfoundland, St. John’s, Newfoundland, Canada

## Abstract

Sarcoidosis with manifest cardiac involvement typically presents with heart failure, conduction abnormalities, or ventricular arrhythmias. Here, we present a case of a young woman whose presentation raised suspicion for metastatic cardiac disease of unknown primary origin. Further investigation revealed cardiac sarcoidosis with multiple intramyocardial granulomatous masses in the absence of significant enlargement of hilar or mediastinal nodes. This case highlights the following: (i) sarcoidosis can mimic metastatic cardiac tumours; and (ii) hilar and mediastinal lymph nodes can be metabolically active in cardiac sarcoidosis in the absence of significant enlargement.

## **Résumé**

La sarcoïdose avec atteinte cardiaque patente se manifeste typiquement par une insuffisance cardiaque, des troubles de la conduction ou des arythmies ventriculaires. Nous présentons ici le cas d’une jeune femme dont le tableau clinique évoquait une pathologie cardiaque métastatique d’origine primitive inconnue. Des examens supplémentaires ont révélé une sarcoïdose cardiaque associée à de multiples granulomes intramyocardiques, en l’absence d’hypertrophie importante des ganglions hilaires ou médiastinaux. Ce cas illustre les points suivants : (i) les symptômes de la sarcoïdose peuvent imiter ceux de tumeurs cardiaques métastatiques; et (ii) les ganglions lymphatiques hilaires et médiastinaux associés à une sarcoïdose cardiaque peuvent présenter une activité métabolique en l’absence d’hypertrophie importante.

Sarcoidosis with clinically evident cardiac involvement typically presents with heart failure, conduction abnormalities, or ventricular arrhythmias.^1^ Sarcoidosis presenting as a single intramyocardial mass mimicking a cardiac tumour is uncommon.^2^ On review of previously reported cases, this case appears to be an extremely rare instance of multiple masses mimicking tumours. This case highlights the utility of multimodality imaging and biopsy in the work-up of this unusual presentation.

## Case

A previously healthy 35-year-old woman was referred following multiple emergency visits for palpitations and a mild but persistent elevation in high-sensitivity troponin level (23.6-33.6 ng/L; normal, 0-15 ng/L). At this visit, she also complained of new chest discomfort, and a left posterior fascicular block was noted on electrocardiogram.

**Figure 1:**
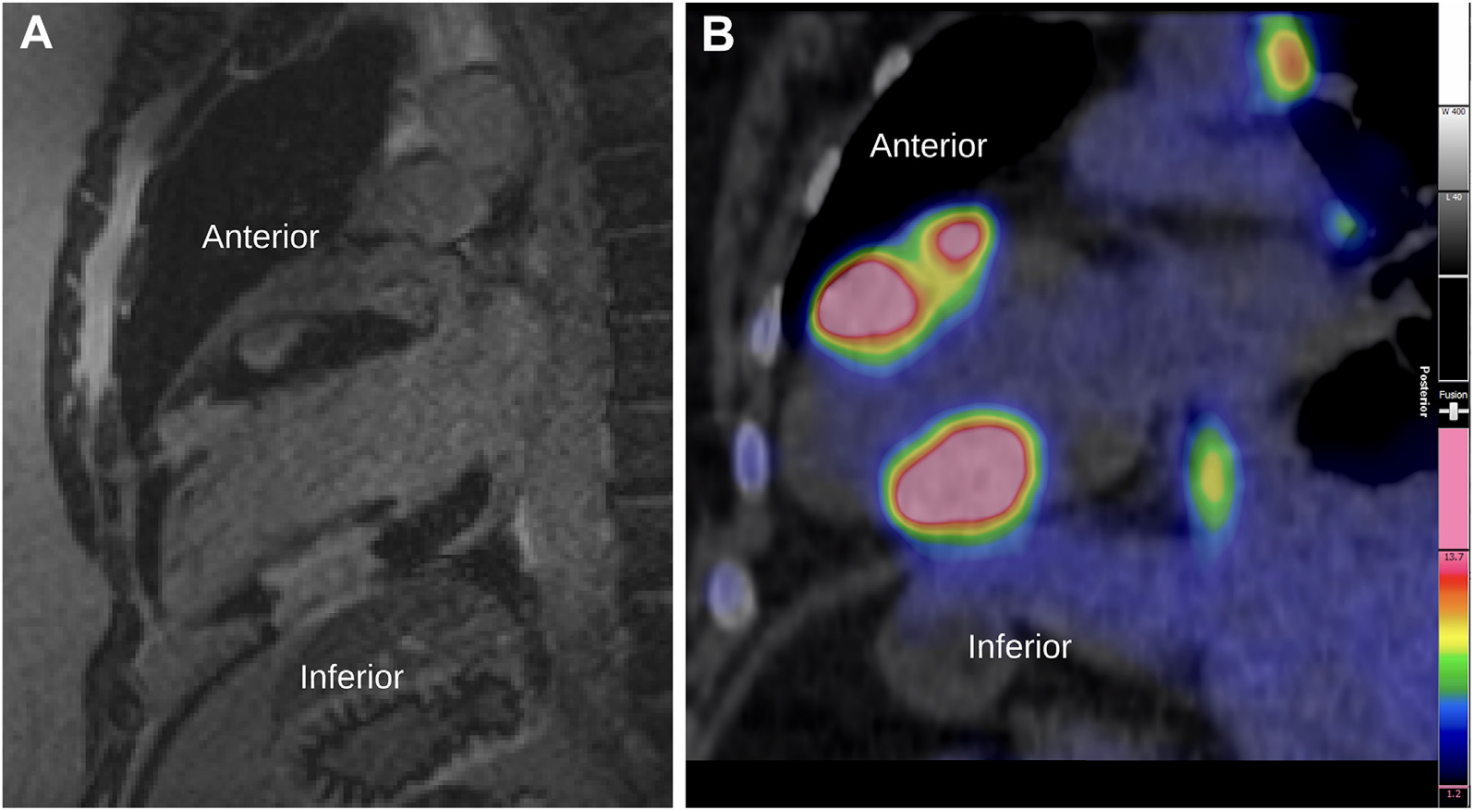
Comparison sagittal views of masses in the inferior wall and anterior wall (**A**) on late-gadolinium enhancement images on magnetic resonance imaging with (**B**) intense ^18^F-fluorodeoxyglucose uptake on cardiac positron emission tomography.

**Figure 2:**
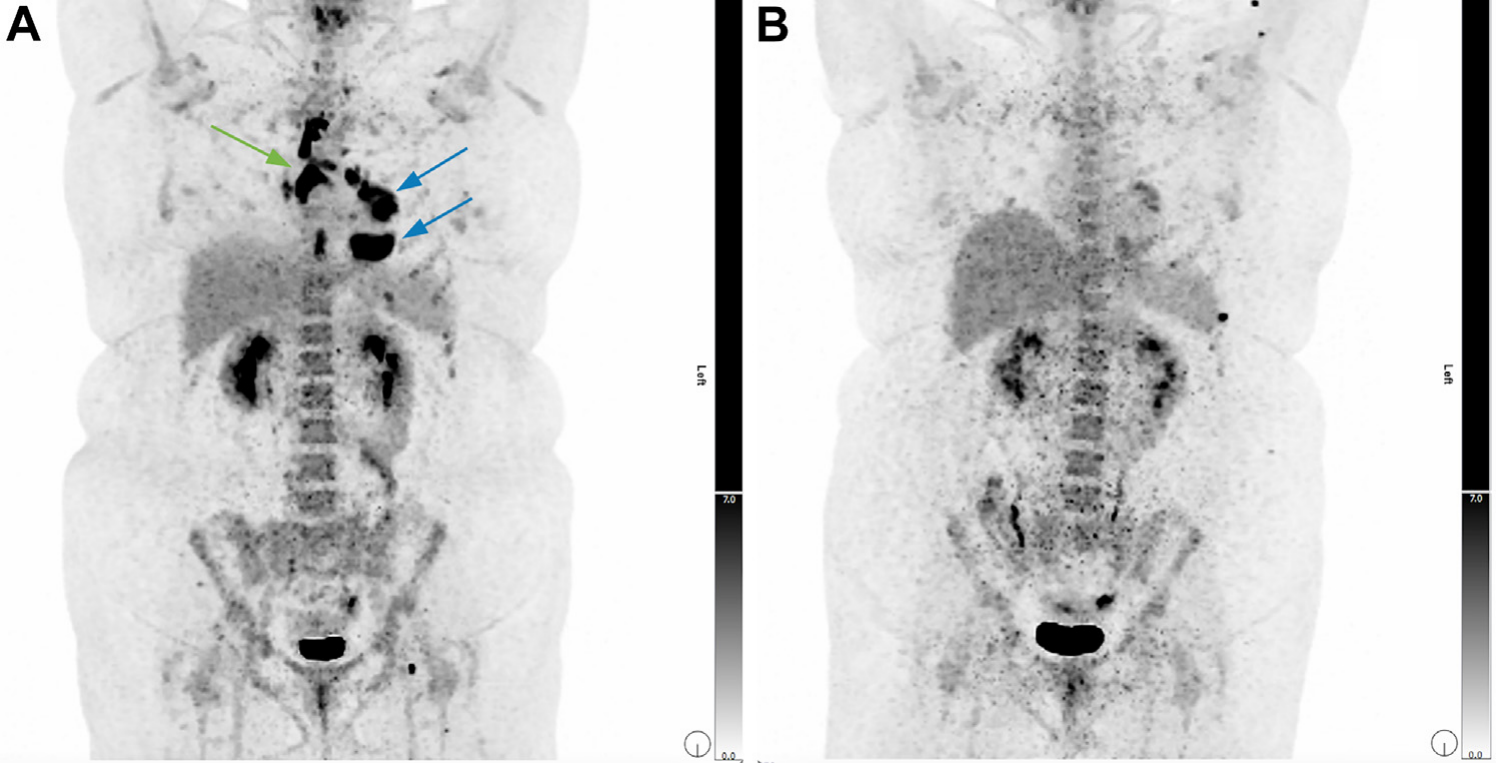
Whole-body positron emission tomography images (**A**) pretreatment, demonstrating cardiac mass (**blue arrows**) and hilar lymph node (**green arrow**) ^18^F-fluorodeoxyglucose uptake. (**B**) Follow-up image at 3 months following treatment with high-dose prednisone.

This presentation prompted suspicion for myocarditis, and cardiac magnetic resonance (CMR) imaging was arranged. CMR imaging revealed infiltrative mass lesions in the mid-left ventricle's anterior and inferior walls on cine imaging up to 4.0 x 2.3 cm, demonstrating late gadolinium enhancement (LGE). An echocardiogram demonstrated an inferior wall motion abnormality with heterogenous Definity contrast (Lantheus, Billerica, MA) uptake in the inferior wall. These masses were initially thought to represent metastatic disease or multiple primary cardiac tumours. Contrast-enhanced computed tomography (CT) of the chest, abdomen, and pelvis was performed to assess for malignant origin; however, this imaging was unremarkable. Repeat CMR imaging with mass protocol revealed a total of 3 lesions. These were characterized by isointensity to surrounding myocardium on T1, mild hyperintensity on T2 fat-sat, and hypointensity with ring-enhancement on perfusion imaging. There were no enlarged hilar or mediastinal nodes noted on the initial CT or CMR images. ^18^F-fluorodeoxyglucose positron emission tomography (FDG-PET) was arranged to search for malignancy and metabolic activity within the lesions. The American Society of Nuclear Cardiology (ASNC) myocardial suppression protocol was followed for all scans. Cardiac FDG-PET demonstrated mass-like foci of intense activity in areas of the left ventricle corresponding to CMR findings of LGE (Fig. 1). A comparison image for T2STIR on magnetic resonance imaging is shown in Supplemental Figure S1. In addition to the cardiac lesions, tracer uptake was seen in mediastinal, hilar, and supraclavicular lymph nodes, as well as splenic involvement not previously noted on prior imaging (Fig. 2A). Endobronchial ultrasound-guided biopsy of the subcarinal lymph node collection showed rare multinucleated giant cells and epithelioid histiocytic collection suggestive of granuloma.

The patient was started on high-dose prednisone, and an implantable cardioverter-defibrillator was placed. In short-term follow-up, her symptoms had resolved, and repeat 3-month FDG-PET showed near resolution of uptake in the cardiac lesions (Fig. 2B).

## Discussion

Autopsy studies show that 25% of patients with sarcoidosis have cardiac involvement, but only about 5% express clinical manifestations. These typically include conduction abnormalities, ventricular arrhythmias, or heart failure.^1^

The diagnosis of cardiac sarcoidosis (CS) can be challenging, especially with limited or no extracardiac involvement. Given that this uncommon disease can masquerade as other entities, multimodality imaging is indispensable. In our case, initial CMR found masses suggestive of primary or metastatic cardiac tumours, but CT found no extracardiac disease. Subsequent FDG-PET discovered cardiac and extracardiac enhancement with intensely active hilar lymphadenopathy. This finding is unusual given that no significant lymph node enlargement was seen on CMR or CT. In circumstances in which sarcoidosis is being considered, such as a young patient with heart block, a CT for lymphadenopathy is often used as a "rule out" test for patients with heart block to exclude cardiac sarcoidosis (CS) as the cause of their heart block. This case supports the notion that high-resolution CT may not be sufficient to rule out CS, as noted by Birnie et al. in 2016.^1^

The “Heart Rhythm Society Expert Consensus Statement on the Diagnosis and Management of Arrhythmias Associated With Cardiac Sarcoidosis” describes 2 pathways for diagnosing CS.^3^ The first is via myocardial tissue demonstrating non-caseating granulomas in the absence of an alternative cause.^3^ The second pathway is a clinical diagnosis from invasive and noninvasive studies. In this pathway, CS is probable if histologic evidence of extracardiac sarcoidosis is present, other cardiac causes have been excluded, and one or more specific findings are present.^3^ Three of these findings are derived from imaging—patchy uptake on dedicated cardiac PET, LGE on CMR, and positive gallium uptake.^3^ In our case, diagnostic criteria were met for probable CS via the second pathway, but several imaging modalities were required to determine whether the lesions were consistent with CS.

In conclusion, our case illustrates an unusual presentation of CS with myocardial involvement mimicking multiple cardiac tumours, and it underscores the importance of multimodal imaging and biopsy.

**Novel Teaching Points**

• Multiple intracardiac tumours mimicking metastatic disease is a rare clinical manifestation of sarcoidosis.
• Hilar and mediastinal lymph nodes can be metabolically active on cardiac PET in the absence of significant enlargement.
• High-resolution CT chest or CMR imaging may not be sufficient to rule out CS.
