# Supplementary material for: Multiple Intramyocardial Masses in an Otherwise Healthy 35-Year-Old Woman
Source: CJC Open. 2021 Dec 24;4(4):432–4. doi: 10.1016/j.cjco.2021.12.009 (PMC9039576; doi:10.1016/j.cjco.2021.12.009)
Supplement: Supplemental Figure S1 [file mmc1.pdf]

Supplementary Material

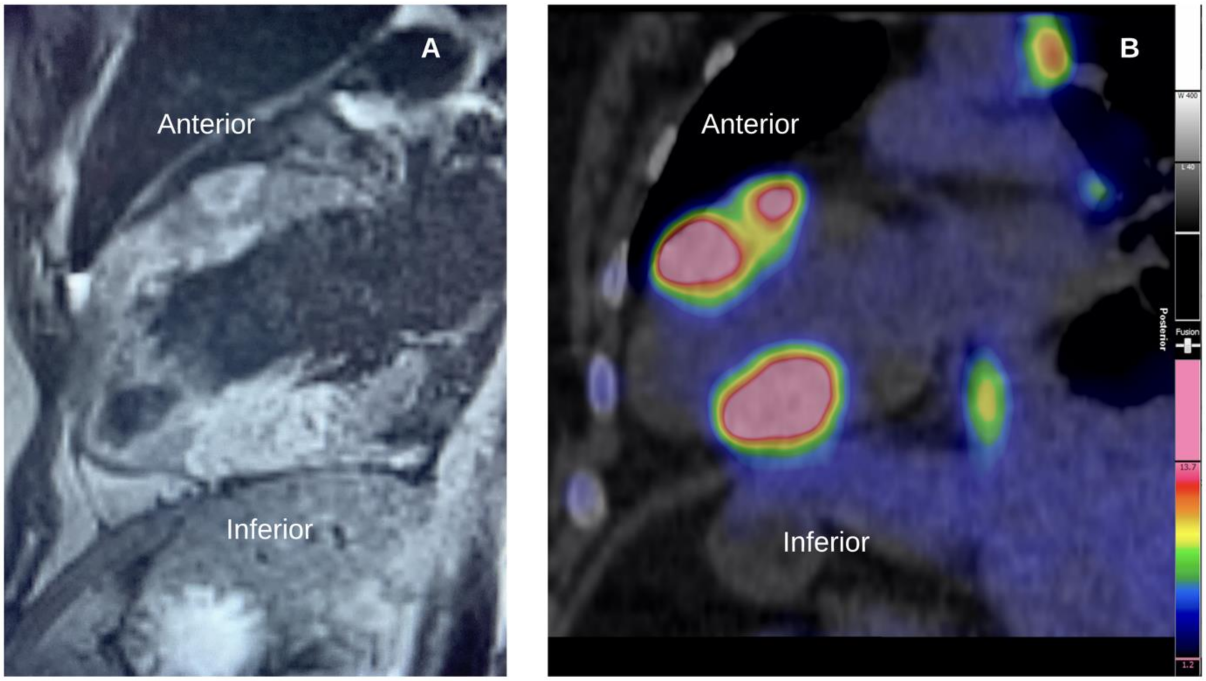

Supplemental Figure S1. Comparison sagittal view of masses in the inferior wall and anterior wall on T2 STIR images on MRI (Panel A) with intense FDG uptake on cardiac PET (Panel B)**.**
